# Gastrointestinal Bleeding Secondary to Metastatic Duodenal Choriocarcinoma in a Patient with Concomitant Peptic Ulcer Disease

**DOI:** 10.1155/2021/6664147

**Published:** 2021-03-02

**Authors:** Ahmed Elfiky, Asmaa Mokhtar, Mira Alsheikh, Hassan Almoussawi, Stephen Mulrooney

**Affiliations:** ^1^Department of Internal Medicine, Staten Island University Hospital-Northwell Health, New York, NY, USA; ^2^Department of Gastroenterology, Staten Island University Hospital-Northwell Health, New York, NY, USA

## Abstract

Testicular tumors are one of the most common solid tumors in young males. Choriocarcinoma usually presents as metastatic disease. Gastrointestinal tract involvement is rare. We report a case of a 40-year-old male presenting to our hospital with a three-day history of dyspnea on exertion and black stool after recent diagnosis of testicular choriocarcinoma. Urgent EGD performed revealed small clean-based fundal ulcer and an antral ulcer without the stigma of recent bleeding. Capsule endoscopy was performed and revealed a bleeding ill-defined mass in the proximal duodenum. A subsequent push enteroscopy showed an ulcerated bleeding mass in the third part of the duodenum that was treated with a hemospray with adequate hemostasis. Pathology was consistent with pure choriocarcinoma. The patient received a cisplatin-based chemotherapy regimen. The patient tolerated the chemotherapy regimen well and was discharged for outpatient follow-up. At the three-month follow-up, the patient did not show evidence of recurrent gastrointestinal bleeding.

## 1. Introduction

Testicular tumors are one of the most common solid tumors in males aged 15–35 years, with germ cell tumors being the most common type [1]. Choriocarcinoma, the most aggressive subtype, usually presents with metastasis owing to the frequent hematogenous and lymphatic spread. The most common sites of metastasis are the lungs, liver, brain, and bone [2]. Gastrointestinal tract involvement is rare. We report a case of a 40-year-old male presenting with upper gastrointestinal bleed secondary to metastatic duodenal choriocarcinoma.

## 2. Case Description

A 40-year-old male presented to the hospital with a three-day history of dyspnea on exertion and black stool. He was recently diagnosed with testicular choriocarcinoma and lung metastasis. He underwent left radical orchiectomy ten days prior to admission. The pathology revealed choriocarcinoma with immunopathological stain positive for human chorionic gonadotropin. The patient was in the process of evaluation for chemotherapy at the time of the presentation. Vital signs upon presentation were notable for a heart rate of 122/minute, otherwise stable. Physical exam was significant for mild epigastric tenderness and melena on the digital rectal exam. Initial blood work revealed acute microcytic anemia with a hemoglobin of 8.4 g/dL. Hemoglobin three weeks before the presentation was 14 g/dL. Upper gastrointestinal bleeding was suspected; the patient was resuscitated and started on pantoprazole infusion. Urgent esophagogastroduodenoscopy (EGD) performed after 10 hours of presentation with the finding of one small clean based fundic ulcer and a 2 cm antral ulcer without the stigma of recent bleeding.

Because of persistent melena and a drop in the hemoglobin level requiring transfusion of 2 pRBCs, colonoscopy was performed with unremarkable finding. Capsule endoscopy was pursued to look for other possible etiologies. It revealed a bleeding ill-defined mass within 3 minutes from the first duodenal image consistent with proximal small bowel bleed.

**Figure 1:**
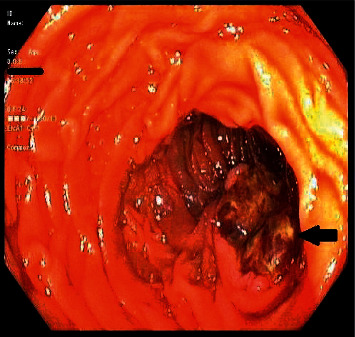
Arrow pointing at a friable ulcerated mass in the third portion of the duodenum.

A subsequent push enteroscopy revealed a friable ulcerated bleeding mass in the third portion of the duodenum that was treated with a hemospray with adequate hemostasis (Figure 1).

**Figure 2:**
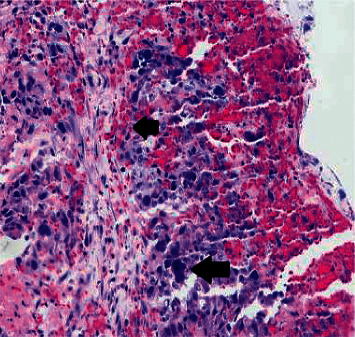
Upper arrow: clusters of cytotrophoblast and hemorrhage; lower arrow: syncytiotrophoblast.

**Figure 3:**
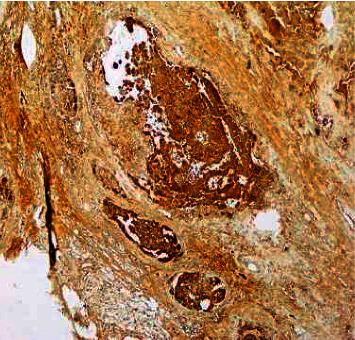
Human chorionic gonadotropin-positive immunohistochemical stain.

The patient received a total of 8 units of packed RBCs during the hospital stay of 10 days. Pathology from enteroscopy was consistent with pure choriocarcinoma, and the immunohistochemical stain is positive for human chorionic gonadotropin (Figures 2 and 3).

Laboratory tests revealed serum human chorionic gonadotropin 516139 mIU/ml [<1, mIU/ml] and alpha-fetoprotein 1.83 ng/ml [<8.3 ng/ml]. The patient started a chemotherapy regimen with etoposide, ifosfamide, and cisplatin. Bleeding was controlled acutely by hemospray and completely ceased after beginning chemotherapy. The patient tolerated the chemotherapy regimen well and was discharged for outpatient follow-up. At the three-month follow-up, the patient did not show evidence of recurrent gastrointestinal bleeding.

## 3. Discussion

Germ cell tumor is the most common testicular cancer. The most aggressive and least common type of germ cell tumor is choriocarcinoma [3]. Less than 8% of testicular germ cell tumors contain a component of choriocarcinoma, and less than 1% of the primary testicular germ cell tumors are pure choriocarcinoma [4].

Testicular tumors usually present as painless testicular mass or swelling. However, in choriocarcinoma, widespread hematogenous dissemination occurs early, and many patients present with metastatic disease [5]. Measurement of serum tumor markers human chorionic gonadotropin and alpha-fetoprotein can be helpful as they are elevated in roughly 80% of choriocarcinoma cases.

A definite diagnosis of choriocarcinoma requires a radical inguinal orchiectomy. The determination of metastatic choriocarcinoma can be challenging as extensive tissue sampling and careful microscopic examination are essential. The most important pathologic characteristic of choriocarcinoma is the coexistence of both syncytiotrophoblast and cytotrophoblast cells, which distinguishes this tumor from other germ cell tumors with only scattered syncytiotrophoblast. Tumor markers can be of great help in establishing the diagnosis. GATA3 is a transcription factor emerging as a sensitive immune marker for choriocarcinoma [6].

Gastrointestinal tract metastasis occurred in 5% of germ cell tumors. The small intestine, most commonly the duodenum, is the most frequent site for metastasis (72%) followed by the esophagus, stomach, and colon [7]. Small bowel involvement manifests as intestinal obstruction or gastrointestinal bleeding, as choriocarcinoma is a highly vascular tumor. Rosenblatt et al. described the first case of severe upper gastrointestinal bleed secondary to metastatic testicular tumor [8].

Treatment of testicular choriocarcinoma depends on the disease stage. Radical orchiectomy of the affected testis and dissection of the affected lymph nodes are the treatment of early-stage disease. Metastatic disease is usually treated with cisplatin-based chemotherapy. The serum level of human chorionic gonadotropin is used to monitor response to treatment [9].

Acute treatment modalities of the bleeding gastrointestinal metastasis are similar to other bleeding etiologies and include endoscopic intervention, embolization, or surgical resection. Bain et al. presented a case of bleeding duodenal choriocarcinoma that was treated with angiography and embolization [10]. Iglesias Gasrcia et al. also presented another case that required surgical intervention for achieving hemostasis after failure of endoscopic injection with adrenaline and arogon plasma coagulation [11]. Bleeding control is usually consolidated by chemotherapy. In our case, despite finding two ulcers on the EGD, high clinical suspicion for separate pathology was required to pursue capsule endoscopy. Despite being a rare presentation, the metastatic bleeding tumor should be considered in a patient with testicular tumors presenting with gastrointestinal bleeding.
